# Supplementary material for: Effects of blood parasite infection and innate immune genetic diversity on mating patterns in a passerine bird breeding in contrasted habitats
Source: PeerJ. 2018 Nov 23;6:e6004. doi: 10.7717/peerj.6004 (PMC6254242; doi:10.7717/peerj.6004)
Supplement: Supplemental Information 1 [file peerj-06-6004-s001.pdf]

Supplementary materials for: Effects of blood parasite infection and innate immune genetic diversity on mating patterns in a passerine bird breeding in contrasted habitats

Dany Garant[1], Audrey Bourret[1], Clarence Schmitt[2], Audrey Turcotte[1], Fanie Pelletier[1], Marc Bélisle[1]

[1] *Département de Biologie, Université de Sherbrooke, 2500 Boulevard de l'Université, Sherbrooke, QC J1K 2R1, Canada*

[2] *Sorbonne Université, CNRS, IRD, INRA, Institut d'Ecologie et des Sciences de l'Environnement de Paris, iEES-Paris, Paris,* 75005, *France*

Supplementary results.

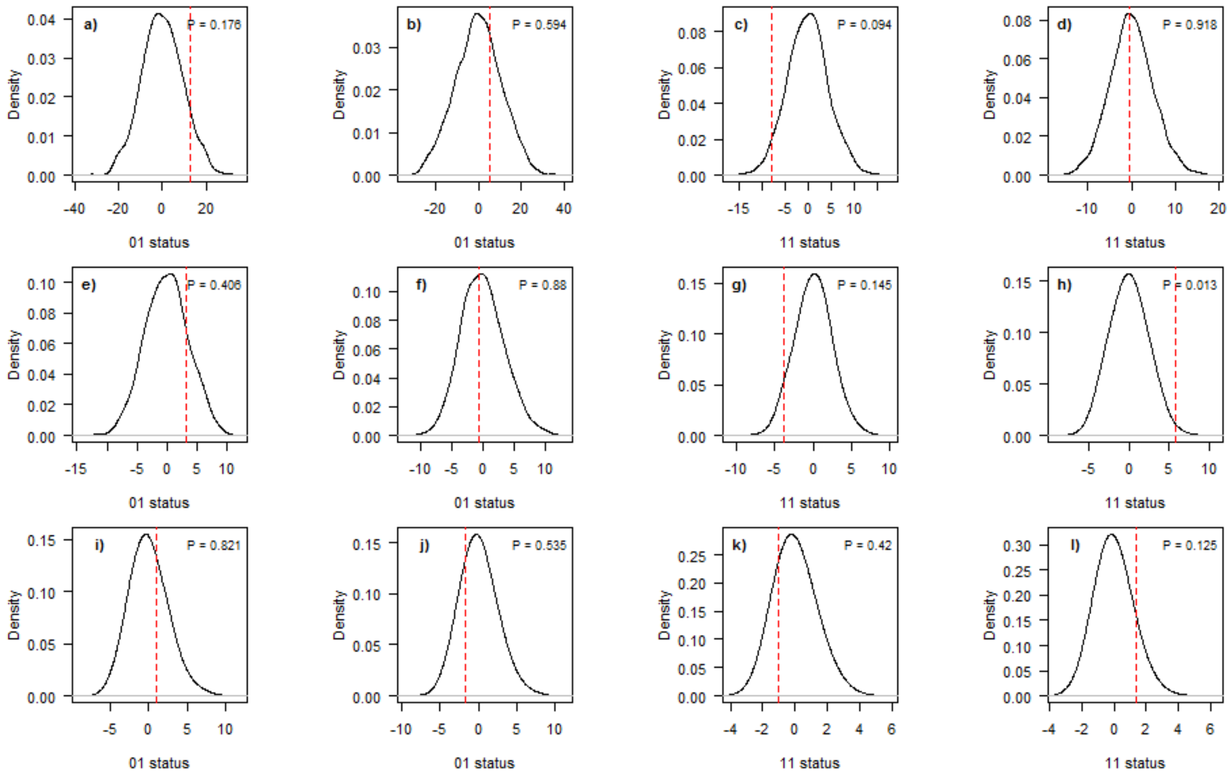

Figure S1. Comparison of the observed numbers of mating pairs of a given status (dashed red line) to the distribution of 1000 random expectations for parasite infection (a-d), AvBD2 (e-h) and AvBD7 (i-l). Results are shown for non-infected/homozygous females paired with infected/heterozygote social

males (a,e,i) and genetic males (b,f,j), and for infected/heterozygote females paired with infected/heterozygote social males (c,g,k) and genetic males (d,h,l) (results for complementary pairs are not shown). P-values, for differences between observed and random expectations, were calculated using two-tailed distributions.

It should be noted that additional analyses conducted at the within habitat level-revealed that intensive habitat, only a marginally non-significant tendency for infected females to pair with infected genetic males more often than random expectations was detected (social males: P = 0.19; genetic males, P = 0.07; all others P-values > 0.14).

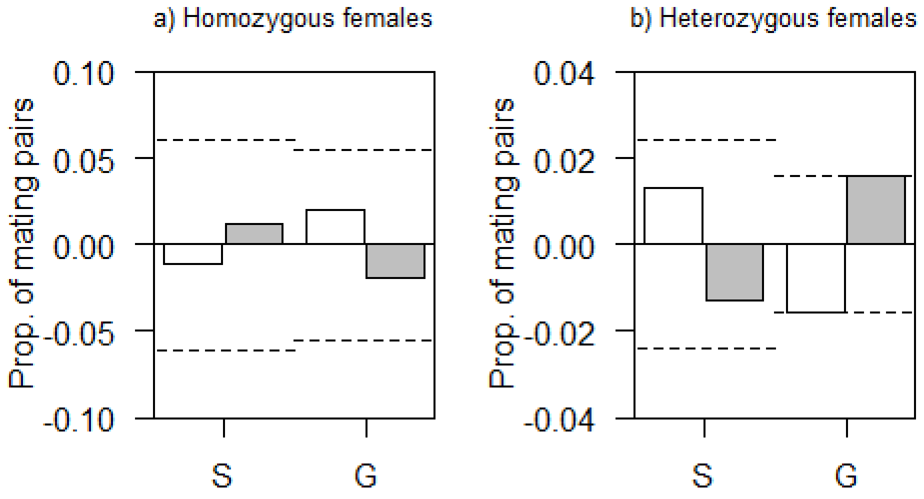

Figure S2. Difference in proportions of observed and expected mating pairs. a) Homozygous females at AvBD7 locus and b) heterozygote females at AvBD7, paired with either social males (S) or genetic males (G). White bars represent homozygous males, and grey bars, heterozygote males. Significance threshold (P<0.05) in each case is represented by a dashed line.

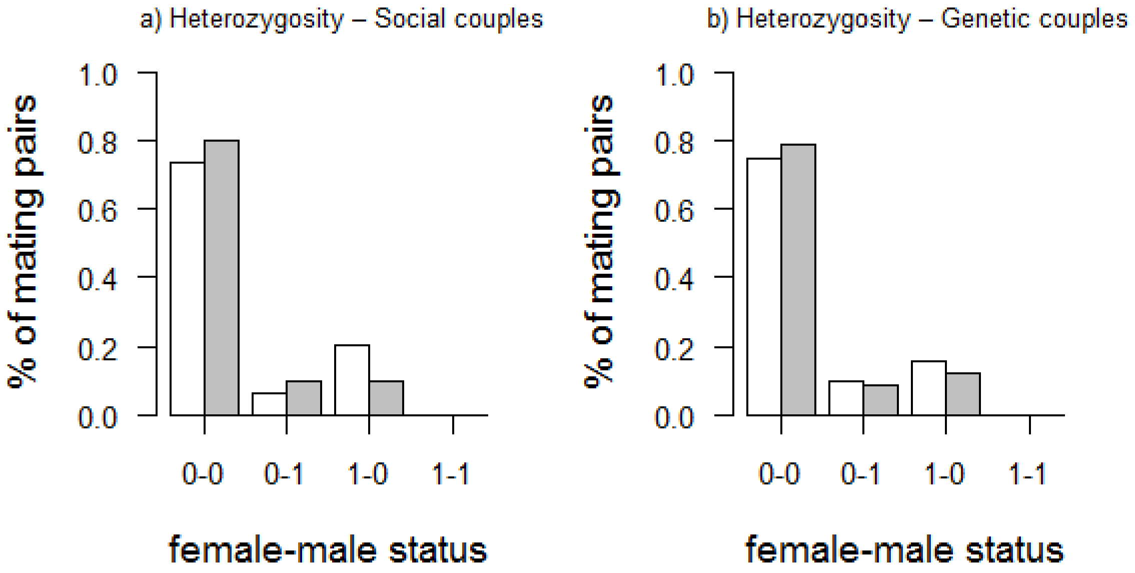

Figure S3. Proportions of observed mating patterns between habitats (white bars: intensive; grey bars: non-intensive) as function of heterozygosity at AvBD7 locus (homozygote: 0, heterozygote: 1), for social couples (a) and genetic couples (b).
